# Development of low-cost electrical stimulation device to promote hiPSC-CM differentiation and functionality

**DOI:** 10.1063/5.0269538

**Published:** 2026-01-02

**Authors:** Nikhith Kalkunte, Sogu Sohn, Cody Callahan, Talia Delambre, Nima Momtahan, Sarah Meng, Amy Brock, Janet Zoldan

**Affiliations:** Department of Biomedical Engineering, The University of Texas at Austin, 107 W Dean Keeton St 3.314, Austin, Texas 78712, USA

## Abstract

Human induced pluripotent stem cell differentiated cardiomyocytes (hiPSC-CMs) hold great potential to resolve cardiovascular disease but are stymied by their functional immaturity. The complex electric potentials measured during cardiogenesis point to the potential of exogenous electrical stimulation in improving cardiac differentiation and functionality. Herein, we create, validate, and implement a low-cost electrical stimulation device to stimulate human induced pluripotent stem cells during cardiac differentiation. Notably, our open-source device enables the generation of dynamic electrical stimulation regimens that may vary in frequency and pulse duration over time. Our results show that cardiac differentiation under dynamic electrical stimulation improves cardiac differentiation efficiency, beating synchronicity, and intracellular calcium handling and flow but impedes contraction compared to static electrical stimulation and no stimulation controls. We also show that pulse duration is an important stimulation parameter to optimize for hiPSC-CM differentiation and functionality. Across nearly all measured metrics, hiPSC-CMs subjected to dynamic pulse duration stimulation during differentiation outperformed those generated under dynamic frequency stimulation. We anticipate that more complex dynamic electrical stimulation regimens may be generated to further optimize hiPSC-CM functionality and maturity.

## INTRODUCTION

Human induced pluripotent stem cell differentiated cardiomyocytes (hiPSC-CMs) hold great potential to improve the clinical outcomes of myocardial infarction. Yet, their translation to the clinic is stymied by their functional immaturity as compared to the myocardial tissue they seek to replace. hiPSC-CMs lack a defined structure,^1,2^ generate significantly lower force during contraction,^2,3^ conduct calcium signals more slowly,^4,5^ and are metabolically inefficient^6,7^ compared to healthy myocardium.

Electric fields and their effect on cardiac contraction and signal propagation have been explored extensively to bridge this functional gap. Cardiomyocytes (CMs), as both signal conduits and contractile units, are particularly susceptible to electrical fields generated via exogenous stimulation. Exogenous electric fields may regulate cell behavior via (1) moving charged cytoplasmic elements, (2) modulating transmembrane potential – triggering ion transport across the membrane, and (3) eliciting conformational change and protein aggregation at the membrane surface.^8^ CMs, with high aggregations of voltage-gated ion channels and signaling cascades responsive to changes in transmembrane potential,^9^ are particularly susceptible to exogenous electric fields.

Prior work investigates the effect of electric fields on CM functionality via electrical stimulation (EStim), with partial success. Seminal work conducted by Nunes *et al.* showcased how the EStim of three-dimensional (3D) wire-like assemblies of hiPSC-CMs, termed Biowires, significantly improved ultrastructural organization, conduction velocity, and calcium handling. Stimulated hiPSC-CMs showed a fourfold increase in the percentage of rod-like CMs, a 40%–50% increase in conduction velocity, and increased sensitivity to caffeine compared to non-stimulated controls.^10^ Extending this work into 3D-engineered heart tissues (EHT) demonstrated how electrical stimulation can improve contractile force and calcium handling. EHTs, stimulated after tissue formation, saw more than two times higher contraction forces,^11^ 30% more dense mitochondria,^12^ and positive force-frequency responses^13^ compared to non-stimulated controls. While markedly improved, hiPSC-CMs subjected to EStim still remain functionally immature relative to healthy adult myocardium.^14^

Citing the strong role electric fields play in embryonic left-right patterning^15–17^ and cardiogenesis,^18,19^ the stimulation of pluripotent stem cells (PSCs) during cardiac differentiation has also been investigated, with limited success. Stimulation of 3D embryoid bodies resulted in a 20% increase in the number of spontaneously beating embryoid bodies,^20^ mediated via the generation of reactive oxygen species (ROS).^21,22^ Recent work has shown the stimulation of monolayers of human induced pluripotent stem cell (hiPSCs) for 2h a day during 15 days of cardiac differentiation increased the expression of cardiac transcription factors, increased the expression of connexin 40 and cardiac intercalated disk proteins (nebulin-related-anchoring protein and þ-catenin), and yields hiPSC-CMs that exhibited faster action potential (AP) depolarization, longer intracellular Ca2þ transients, and slower AP duration at 90% of repolarization, resembling fast conducting fibers.^23^ Finally, varying stimulation parameters such as frequency and onset during cardiac differentiation have been shown to promote the differentiation of hiPSCs into either ventricular or atrial CMs.^24^

While promising, prior studies investigating the impact of electrical stimulation during cell differentiation yield cardiomyocytes with significant functional gaps, relative to adult myocardium. We hypothesize that this may be due to the oversimplification of the stimulation cells, which are subjected to during differentiation. Prior studies that apply electrical stimulation during cardiac differentiation do so in a static manner, wherein stimulation parameters are held constant over the whole regimen.^23,25,26^ Dynamic stimulation regimens or regimens wherein stimulation frequency and pulse duration changeover the course of the regimen may more closely mimic electric fields present during embryogenesis. Seminal works document changes in current charge density over mouse and chick embryogenesis.^15,27,28^ Dynamic endogenous electric fields during embryogenesis have been shown to drive the cell migration of the neural crest,^29^ left-right patterning,^30^ and differentiation.^31^ Indeed, subjecting cardiomyocytes to electrical stimulation that increases in frequency at a rate of 0.33 Hz/day improved CM tissue structure and upregulated genes encoding for adult-like signal conduction, energetics, and calcium handling.^12^

Herein, we sought to evaluate the impact of dynamic electrical stimulation on cardiac differentiation and hiPSC-CM functionality. Due to the success seen in the maturation of terminally differentiating cardiomyocytes with dynamic frequency stimulation and the variable electric currents measured during embryogenesis, we suggested that stimulation during differentiation should also be dynamic and sought to test two varying stimulation parameters: frequency and pulse duration.

The recent advances in off-the-shelf, low-cost electronics create ample opportunity for the development of open-source devices that may be modified to generate a large range of dynamic electrical stimulation regimens. Current electric stimulation devices are expensive and do not support open-source modifications for dynamic electrical stimulation. Early systems that studied electrical stimulation in cardiomyocytes were effective but required custom printed circuit board (PCB) instrumentation and one-off-manufactured parts.^10,32^ More recent devices built for cardiomyocyte electrical stimulation leverage off-the-shelf Arduino electronics but do not support the ability to dynamically change electrical stimulation parameters over time.^33–36^ Therefore, there exists a need for open-source equipment, preconfigured to generate a wide variety of dynamic stimulation regimens that may vary over time.

We first constructed and characterized a low-cost electrical stimulation device capable of static and dynamic electric stimulation. Leveraging consumable electronics, we provide an open-source design to support further modification by the community. We employed this device to subject differentiating hiPSCs to static and dynamic electric stimulation regimens at various frequencies and pulse durations.

We found that electric stimulation during differentiation across all regimens improves cardiac differentiation by as much as 100% in the case of dynamic pulse duration regimens. We also show that dynamic stimulation regimens improve beating synchronicity and intracellular calcium handling but can stymie contraction velocity relative to static stimulation regimens. Finally, we see that cell response depends on both regimen variance over time and the varied parameter, as varying frequency and pulse duration result in differences in hiPSC-CM functionality. We identify pulse duration optimization as the most likely to further improve hiPSC-CM maturity.

## RESULTS

### Device characterization

**FIG. 1.**
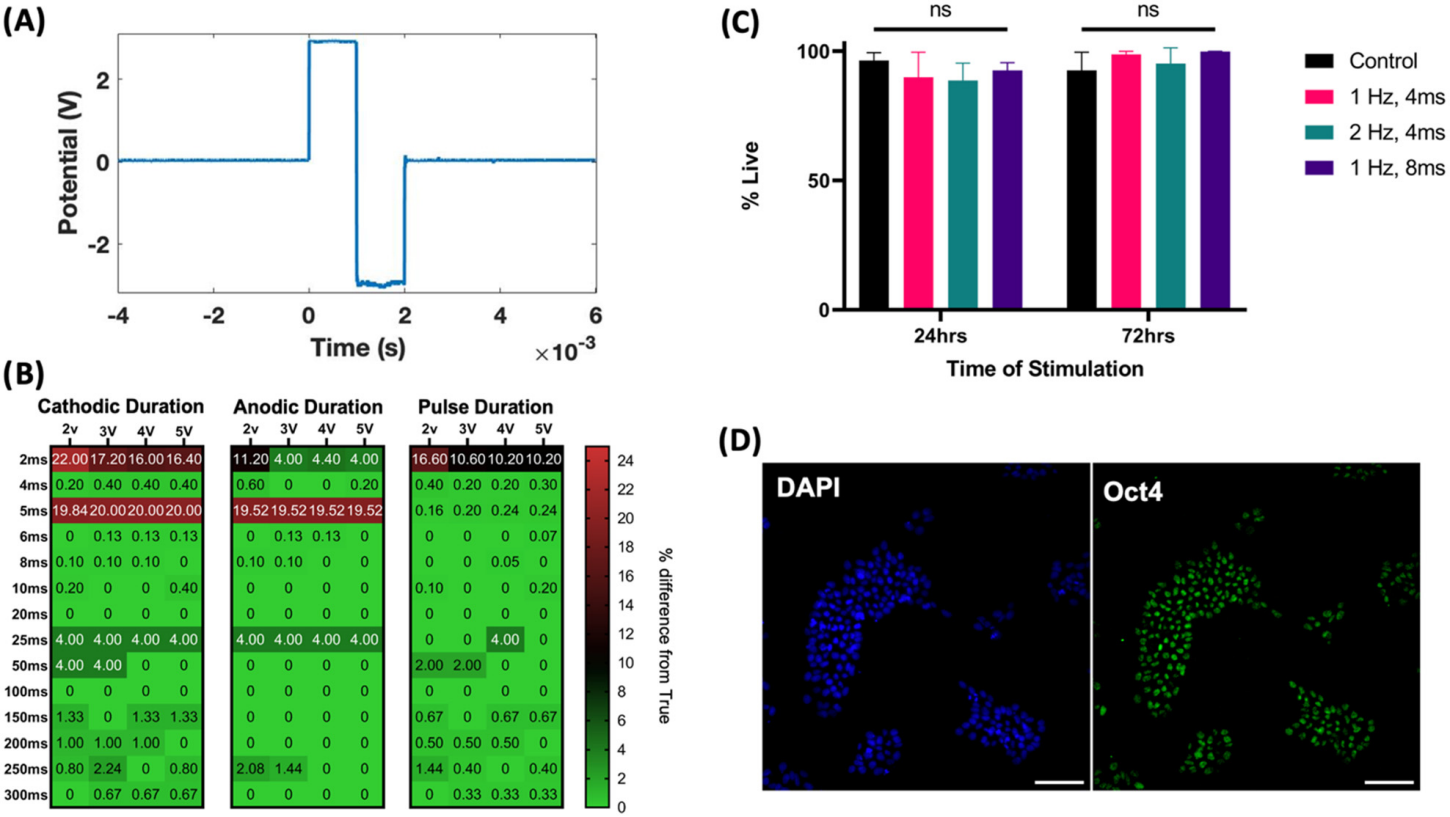
Electrical stimulation signal assessed for signal fidelity and biocompatibility. (a) Oscilloscope recordings of recorded signals demonstrate generation of biphasic, monophasic, or alternating monophasic signal pulses. (b) Signal fidelity of biphasic stimulation is assessed via cathodic duration (duration of positive voltage), anodic duration (duration of negative voltage), and total pulse duration. Percent error of measured pulse parameters compared to expected are assessed across voltages and pulse durations. (c) Minimal cell death is seen in hiPSCs when subjected to biphasic stimulation (4 V, 4 ms) at 1 or 2 Hz over the course of 72 h (n = 6). (d) Representative images of hiPSCs after 72 h of electrical stimulation (1 Hz, 4 v, 4 ms) show nucleus colocation of pluripotency marker Oct. 4. Scale bar = 100 *μ*m.

Oscilloscope recordings of generated signals confirm the generation of biphasic, monophasic, and alternating monophasic pulses [Fig. 1(a), supplementary Data S1]. Signal fidelity was assessed by collecting the generated signal via oscilloscope recording (Tektronix) and calculating the percent error between measured and expected signal parameters. This assessment yielded <5% across all durations at pulse durations greater than 2 ms, with 5 ms being an exception, resulting in a 20% error [Fig. 1(b)]. Electrical stimulation of hiPSCs at a range of pulse durations and frequencies over 72 h resulted in non-significant differences in %live cells compared to no stimulation controls [Fig. 1(c)]. Immunostaining of hiPSCs, post 72 h of stimulation at 1 Hz, 4 ms, 4 V, shows colocalization of OCT-4 transcription factor with DAPI nuclear stain, indicating retention of pluripotency [Fig. 1(d)]. The pulse sensing module was calibrated by creating a calibration curve using oscilloscope readings from a test circuit with known voltage and resistance. Extended testing of the pulse sensing module over 72 h indicates the module has high accuracy in sensing voltage and current (<10% error), while calculated resistance is prone to sensor noise (supplementary Data S2). Measurement of frequency over 10 days of stimulation in frequency ramped stimulation shows linear increases in stimulation frequency at 0.1 Hz per day rate. Similarly, measuring ramped duration over 10 days of stimulation shows a linear increase in pulse duration at 0.4 ms per day (supplementary Data S3).

### Cardiac differentiation

**FIG. 2.**
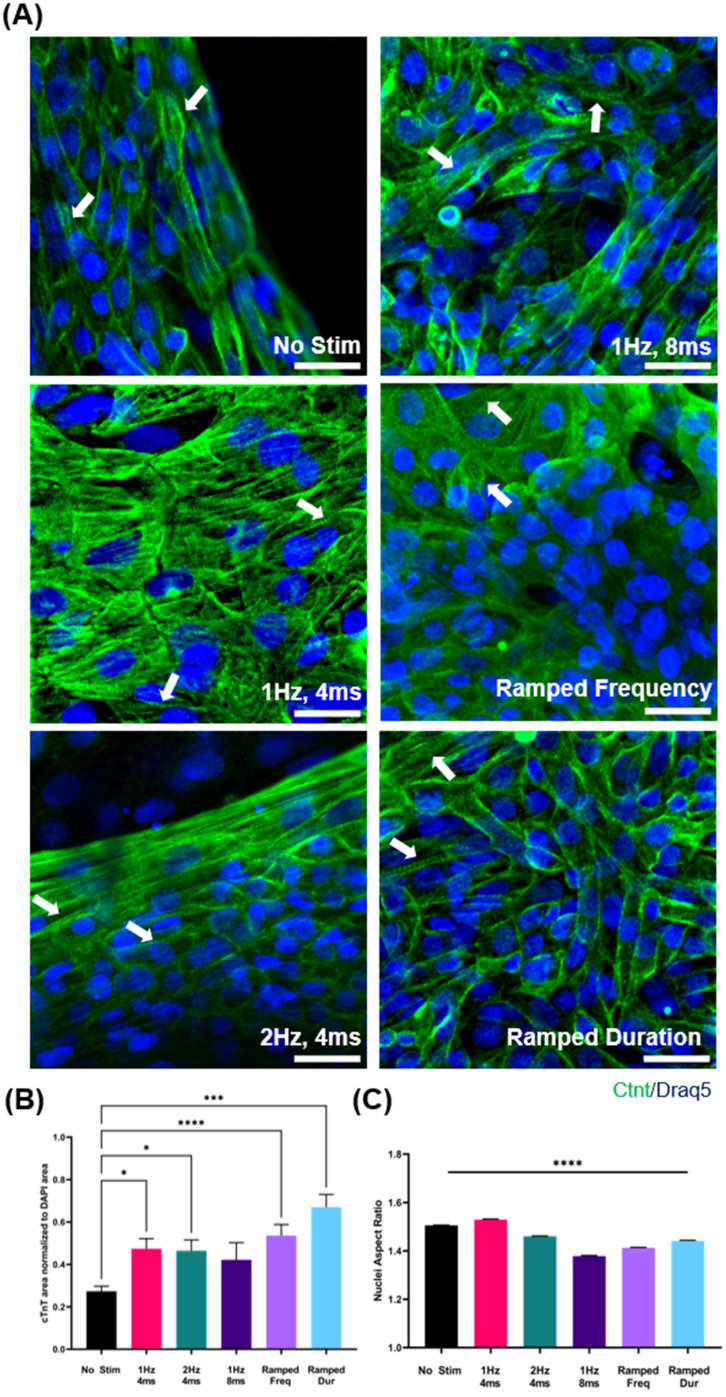
hiPSCs are differentiated under electrical stimulation and are assessed for cardiac purity. (a) hiPSC-CMs differentiated under electrical stimulation are stained for cTnT (green) and nuclei (blue). Sarcomeres are present in all conditions indicated with white arrows. Scale bar = 25 *μ*m. (b) hiPSC-CMs differentiated under electrical stimulation are assessed for cTnT expressions via immunostaining. cTnT area is normalized to DAPI area (n = 20). (c) hiPSC-CMs differentiated under electrical stimulation are assessed for orientation via nuclei aspect ratio (n = 20). ^*^p < 0.05, ^**^p < 0.01, ^***^p < 0.001, and ^****^p < 0.0001.

Electrical stimulation during cardiac differentiation, across all regimens, is shown to significantly increase the percentage of differentiated cardiomyocytes in heterogeneous cell cultures. First, electrical stimulation does not inhibit the generation of functional CMs, as indicated by spontaneous beating and immunostained sarcomeres [Fig. 2(a)]. Next, we see that both static and dynamic electric stimulation result in significantly higher normalized cTnT area than no stimulation controls (0.27 ± 0.02). Comparing static and dynamic, we see that ramped frequency and duration result in a non-significant increase in the enrichment of cardiomyocytes compared to static stimulation. In assessing the impact of stimulation parameter variance, we see that dynamic pulse duration non-significantly increases normalized cTnT area compared to ramping frequency [Fig. 2(b)].

### Cell orientation

Electrical stimulation had mixed effects on cell orientation as measured by the elongation of hiPSC-CM nuclei and cTnT fiber orientation. As an analog of cell elongation, we measured nuclei aspect ratio, wherein a value close to 1 indicates circular nuclei (no elongation) and a value close to 2 indicates elliptical nuclei (elongation). No stimulation hiPSC-CMs are seen to have modest elongation (1.51 ± 0.01). Static stimulation at 1 Hz, 4 ms (1.53 ± 0.01) is seen to significantly increase nuclei aspect, while cells subjected to stimulation at 2 Hz, 4 ms (1.46 ± 0.01) and 1 Hz, 8 ms (1.38 ± 0.01) show significantly lower nuclei aspect ratio. Comparing static and dynamic EStim regimens, we find that subjecting cells to ramped frequency stimulation (1.41 ± 0.01) reduces nuclei aspect ratio compared to static frequency stimulation. In contrast, ramping pulse duration (1.44 ± 0.01) simulation increases nuclei aspect ratio compared to stimulation at static pulse duration. Varying pulse duration during stimulation attenuates nuclei elongation less than varying stimulation frequency [Fig. 2(c)]. Evaluation of cTnT fiber orientation reveals mixed effects of electrical stimulation as well. Cells subjected to 2 Hz, 4 ms (0.19 ± 0.02) stimulation and Ramped Duration (0.27 ± 0.03) regimens show significantly more aligned cTnT fibers as compared to cells subjected to 1 Hz, 4 ms (0.10 ± 0.01), 1 Hz, 8 ms (0.08 ± 0.02), Ramped Frequency (0.08 ± 0.01), and no stimulation controls (0.10 ± 0.01). While cTnT fiber alignment is a hallmark of cardiomyocyte maturity, electrical stimulation across all conditions is shown to only modestly increase cell alignment (supplementary Data S4).

### Electrochemical coupling

Assessment of intracellular calcium transients reveals hiPSC-CMs differentiated under electrical stimulation possess significantly improved calcium handling properties.

#### Beating synchronicity

**FIG. 3.**
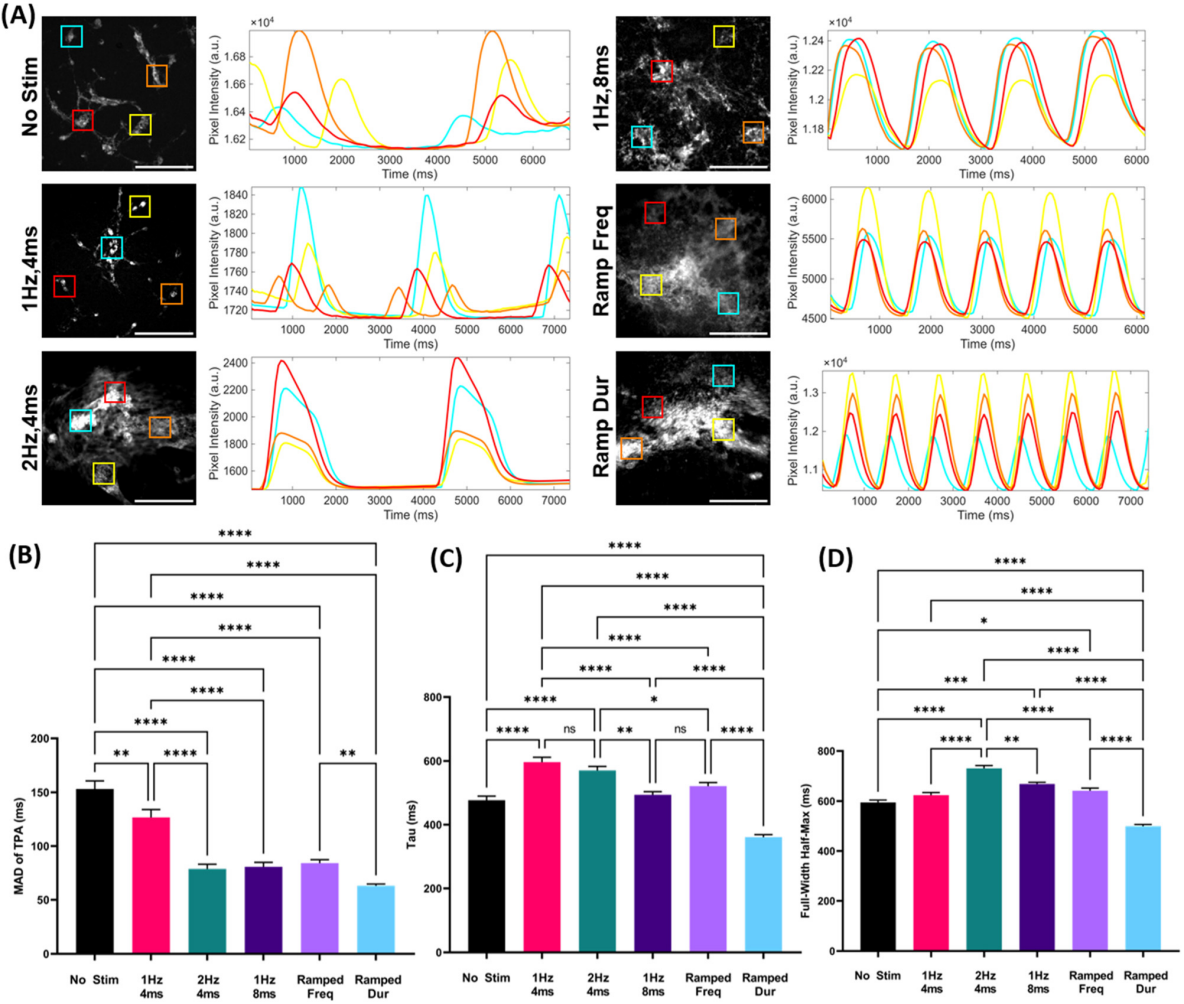
Intracellular calcium handling and synchronicity improve with dynamic frequency stimulation. (a) Representative calcium traces of GCaMP hiPSC-CMs differentiated under electrical stimulation. Scale bar = 500 *μ*m. (b) Synchronicity (n = 215). (c) Calcium cycle duration (n = 175). (d) Decay constant (n = 175) after 10 days of stimulation. ^*^p < 0.05, ^**^p < 0.01, and ^****^p < 0.0001. MAD of TPA, median absolute deviation of time of peak arrival.

We evaluate the degree of synchronous contraction in hiPSC-CMs on day 10 of differentiation via the time of peak arrival median absolute deviation (TPA-MAD) of GCaMP signal. In a synchronized cardiac syncytium, calcium peaks around the same time throughout the tissue, giving a low MAD value, while in less synchronized tissue, peak times vary more, resulting in a higher MAD value. Electric stimulation during differentiation, including both static and dynamic regimens, significantly increases synchronicity as compared to no stimulation controls (153.00 ± 7.57). Static stimulation at 1 Hz, 4 ms (126.70 ± 7.38) provides modest increases in synchronicity compared to no stimulation controls, but the most significant increases to synchronicity come with higher frequency and pulse duration regimens. Stimulation at 2 Hz, 4 ms (78.80 ± 4.35), 1 Hz, 8 ms (80.75 ± 4.05), ramped frequency (84.13 ± 3.23), and ramped duration (63.15 ± 1.61) all results in hiPSC-CMs with significantly higher degrees of synchronicity as compared to no stimulation controls—indicating synchronicity may be driven by higher energy stimulation regimens rather than any difference in static vs dynamic nature of stimulation. Cells differentiated under ramped duration stimulation are seen to have greater degrees of synchronicity than cells subjected to ramped frequency [Fig. 3(b)].

#### Calcium cycle duration

Calcium cycle duration was evaluated via the full-width at half-max (FWHM) of peaks within the GCaMP signal. Maturity correlates with larger FWHM values. Electrical stimulation during differentiation is seen to largely increase calcium cycle duration, relative to no stimulation controls (594.80 ± 9.78 ms). Comparing static and dynamic stimulation regimens, cells differentiated under static 2 Hz, 4 ms (731.5 ± 11.25) and 1 Hz, 8 ms (668.70 ± 7.13) stimulation regimens show significantly longer FWHM as compared to ramped frequency (641.70 ± 10.68) and ramped duration (499.40 ± 7.29). Importantly, ramped duration results in significantly shorter FWHM than ramped frequency [Fig. 3(c)].

#### Calcium reuptake rate

We evaluated the performance of intracellular calcium handling protein machinery by measuring the decay constant (τ) of the measured GCaMP signal. Here, all stimulation regimens show increased decay constant compared to no stimulation controls (476.90 ± 13.09), with the notable exception of ramped duration (360.70 ± 8.01) stimulation. Comparing static and dynamic regimens, we see that cells subjected to ramped regimens show significantly lower decay constants than static regimens. Ramped frequency (521.20 ± 11.15) stimulated cells show lower decay constant as compared to static 1 Hz, 4 ms (596.30 ± 15.58) and 2 Hz, 4 ms (570.40 ± 12.81) regimens. Similarly, ramped duration results in significantly lower decay constants vs cells differentiated under static 1 Hz, 8 ms (491.10 ± 9.50), and notably, ramped duration results in significantly lower τ compared to ramped frequency [Fig. 3(d)].

### Calcium flow velocity and directionality

**FIG. 4.**
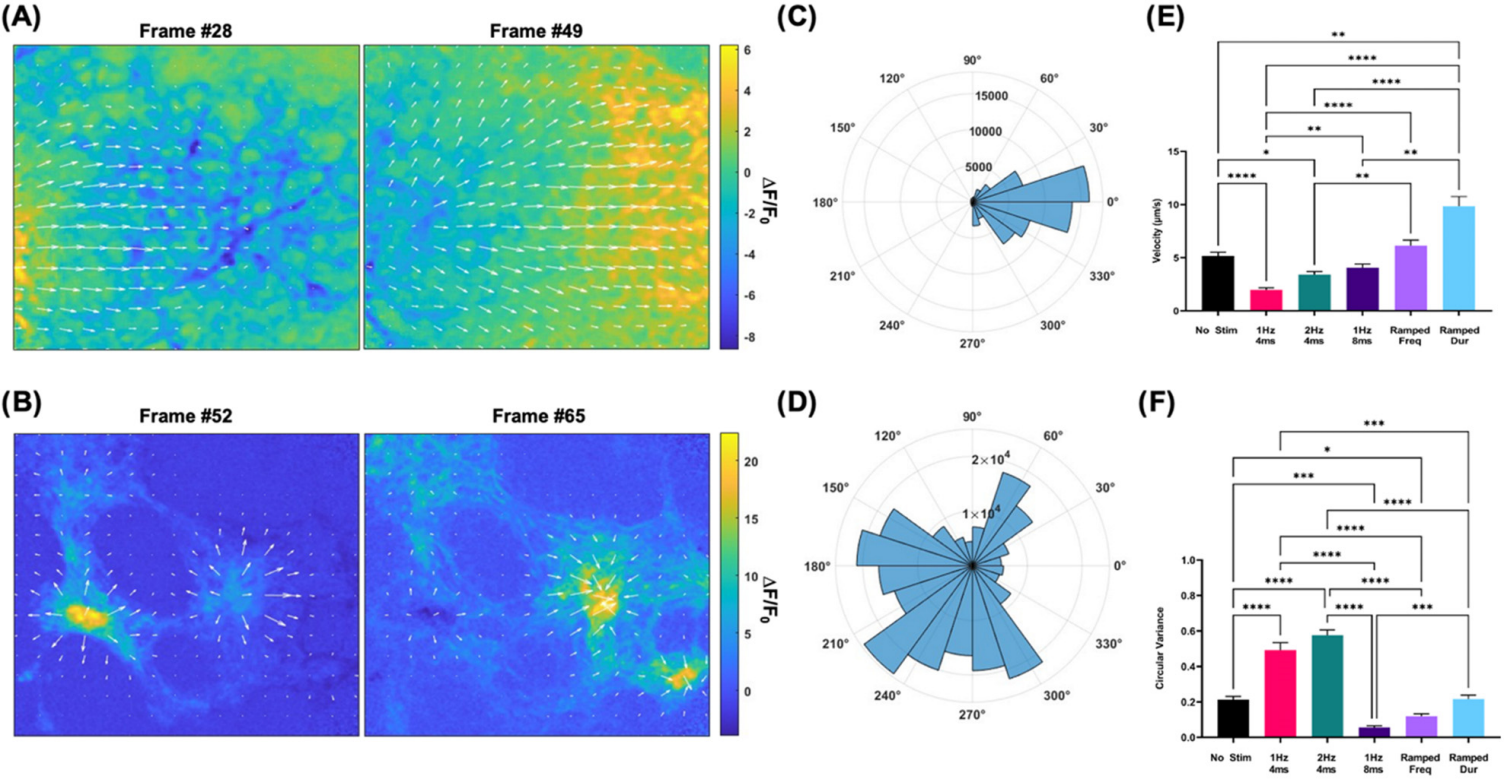
Calcium propagation velocity and directionality improve with dynamic electrical stimulation. (a) Representative images of unidirectional calcium flow annotated with derived flow vectors. Within the 120-frame image sequence taken, frame #28 shows the beginning of a calcium waveform propagation, while frame #49 shows its movement out of the field of view. (b) Representative image sequence of multidirectional calcium flow with derived flow vectors. Vectors show source and sink calcium propagation patterns instead of unidirectional propagation that would indicate efficient and extensive gap junction coupling. (c) Polar rose plot of unidirectional calcium flow vectors derived from the image sequence displaced in (a). (d) Polar rose plot of vectors derived from representative image sequence displayed in (b). The high degree of vector phase dispersion is qualitatively seen, indicating multidirectional calcium propagation. (e) Average vector magnitude of calcium flow is calculated across electrical stimulation conditions (n = 40). (f) Average circular variance of vector phase is calculated across electrical stimulation conditions (n = 45). ΔF/F0. Change in fluorescence normalized to 1 Hz, 4 ms fluorescence. ^*^p < 0.05, ^**^p < 0.01, ^***^p < 0.001, and ^****^p < 0.0001.

Calcium transient flow analysis illustrates electrical stimulation's effect on calcium flow velocity and directionality. Focusing first on calcium flow velocity, static electric stimulation decreases calculated calcium flow, while dynamic stimulation regimens increase it. Cells differentiated under 1 Hz, 4 ms stimulation (1.97 ± 0.20 *μ*m/s) and 2 Hz, 4 ms (3.42 ± 0.27 *μ*m/s) stimulation show significantly reduced calcium flow velocities compared to no stimulation controls (5.16 ± 0.36 *μ*m/s). 1 Hz, 8 ms stimulation (4.04 ± 0.36 *μ*m/s) also leads to decreased calcium flow velocity, relative to control, although these differences are not statistically significant. Switching to dynamic stimulation regimens, both frequency (6.13 ± 0.52 *μ*m/s) and pulse duration (9.85 ± 0.90 *μ*m/s) significantly increase calcium flow velocity relative to static stimulation regimens. Notably, ramped duration stimulation leads to the highest calcium flow velocity, ∼60% higher than ramped frequency stimulation and ∼90% higher than no stimulation controls [Fig. 4(e)].

When assessing calcium flow directionality, static stimulation produces multidirectional calcium flow, while dynamic stimulation results in unidirectional calcium flow. Cells subjected to static 1 Hz, 4 ms (0.49 ± 0.04) and 2 Hz, 4 ms (0.58 ± 0.03) stimulation show significantly increased calcium flow phase variance, relative to no stimulation controls (0.22 ± 0.02), indicating multidirectional flow. 1 Hz, 8 ms stimulation (0.05 ± 0.01) results in a considerable decrease in phase variance relative to control, indicating more unidirectional calcium flow. Comparing static and dynamic regimens reveals different trends across parameters. Within frequency, ramping frequency (0.12 ± 0.01) during differentiation results in cells with lower phase variance, relative to static frequency regimens. In contrast, within pulse duration, cells differentiated under ramped duration stimulation (0.22 ± 0.02) exhibit lower phase variance relative to 1 Hz, 4 ms stimulation but higher phase variance compared to cells differentiated under 1 Hz, 8 ms stimulation. Non-significant differences exist between the phase variance measured in cells differentiated under dynamic frequency and pulse duration stimulations [Fig. 4(f)].

### Cardiomyocyte contractility

Electric stimulation, across all conditions, is shown to decrease contraction/relaxation velocity. Evaluating contraction velocity first, we see cells differentiated under static 1 Hz, 4 ms (27.19 ± 0.82), 2 Hz, 4 ms (35.44 ± 0.81), and 1 Hz, 8 ms (28.09 ± 0.73) stimulation all show significantly decreased contraction velocities compared to no stimulation controls (39.67 ± 0.94). Cells exposed to ramped frequency (23.31 ± 2.94) or pulse duration (23.18 ± 0.78) stimulation regimens both resulted in lower contraction velocities compared to cells exposed to their respective static regimens.

**FIG. 5.**
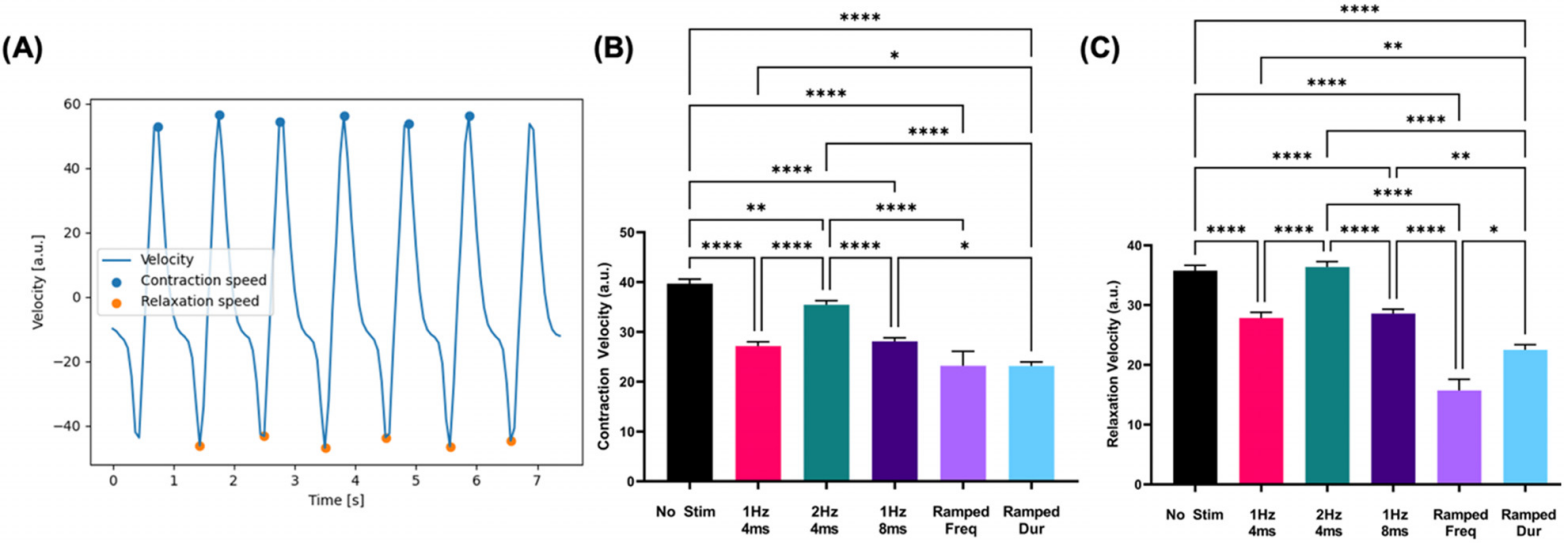
Cardiomyocyte contraction decreases with electrical stimulation. (a) BeatProfiler software was used to process phase contrast image sequences of beating hiPSC-CMs to derive traces of pixel velocity. (a) Plotting velocity magnitude over time allows for the identification and collection of peak contraction and relaxation speed. Average contraction (b) and relaxation (c) velocities are calculated across electrical stimulation conditions and compared to no stimulation controls (n = 135). ^*^p < 0.05,^**^p < 0.01, ^****^p < 0.0001.

Analysis of measured relaxation velocities follows similar trends. Cells subjected to 1 Hz, 4 ms (27.85 ± 0.94) and 1 Hz, 8 ms (28.57 ± 0.73) stimulation exhibited significantly lower relaxation velocity as compared to no stimulation controls (35.78 ± 0.89). 2 Hz, 4 ms (36.37 ± 0.88) stimulation results in relaxation velocities that are not significantly different from those measured in no stimulation controls. Cells differentiated under ramped frequency (23.18 ± 0.78) stimulation showed decreased relaxation velocities compared to 2 Hz, 4 ms regimens. Similarly, ramped duration (22.53 ± 0.84) stimulation led to significantly lower relaxation velocities compared to static duration conditions. Additionally, cells differentiated under ramped duration conditions showed significantly increased relaxation velocities relative to those under ramped frequency conditions (Fig. 5).

### RNA-seq

**FIG. 6.**
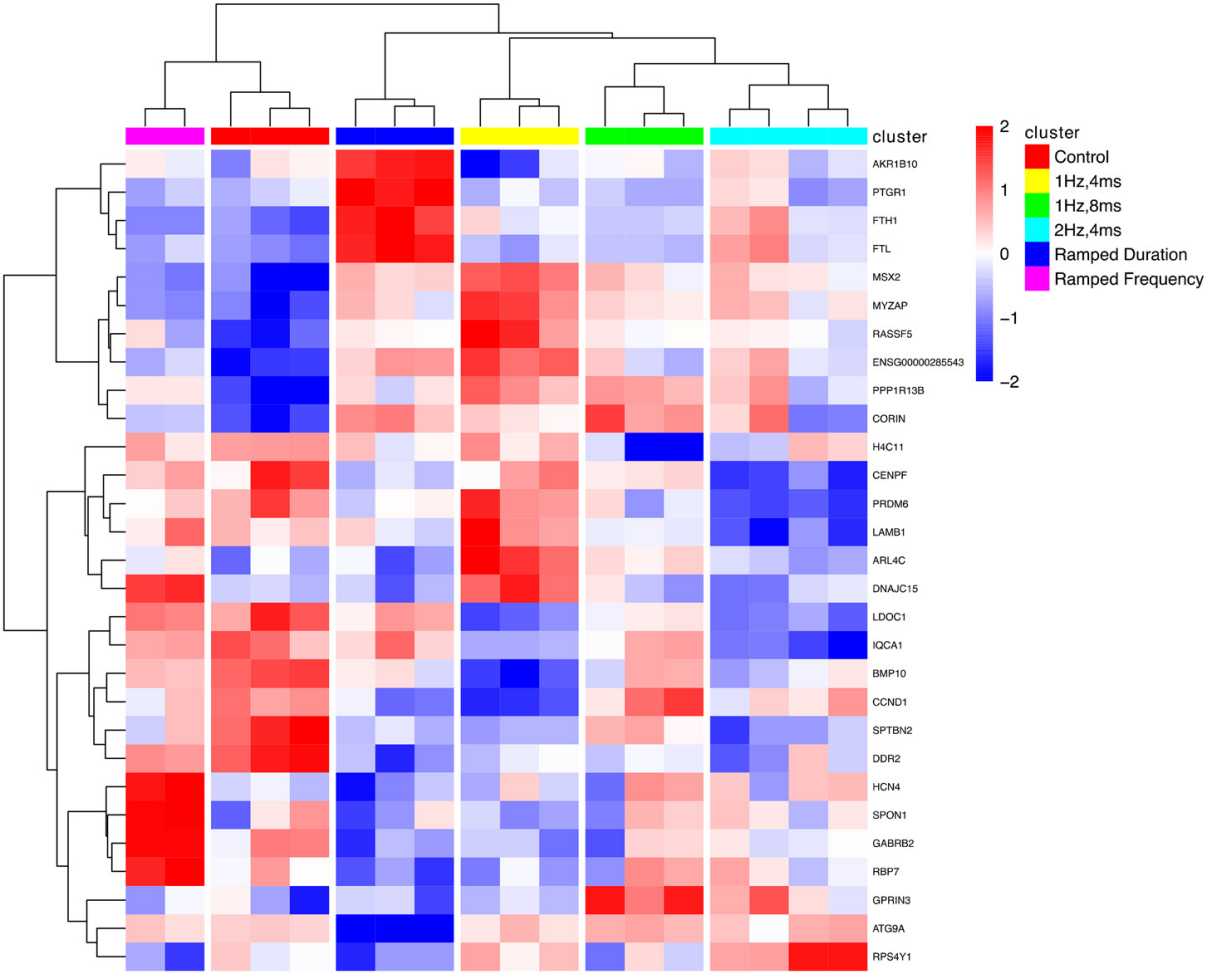
Bulk-RNA Tagseq expression differences between static and dynamic electrical stimulation. Clusterchart of top (p-value < 0.05) log-transformed counts-per-million genes.

RNA from individual differentiations per group was collected and sequenced in two batches; however, due to a strong batch effect, downstream analysis was limited to only the latter samples. Despite this, multidimensional scaling indicated intragroup heterogeneity, as seen in the 2 Hz, 4 ms, 1 Hz, 8 ms, and no stimulation control groups. Interestingly, the ramped duration condition clustered near the 1 Hz, 4 ms group, while the ramped frequency condition clustered similarly to control along dimension 1 (supplementary material, Fig. 4). Investigating at the gene level, via ClusterMap, reveals how EStim significantly impacts gene expression within these clusters. Cells differentiated under ramped frequency show higher expression of *HCN4*, a hyperpolarization activated potassium channel, and similar expression of *BMP10*, which maintains the expression of *NKX2-5*, compared to control.^37,38^ Atrial natriuretic peptide converting enzyme *CORIN* had upregulated expression in cells subjected to ramped duration, 1 Hz, 4 ms, and 1 Hz, 8 ms, as compared to the control. Higher frequency stimulation is seen to downregulate CORIN, as cells differentiated under 2 Hz, 4 ms, and ramped frequency stimulation had downregulated expression compared to cells differentiated under 1 Hz, 4 ms.^39,40^ Finally, ramped duration stimulation had upregulated the expression of intracellular iron storage protein *FTH1* and iron metabolism protein *FTL* compared to other groups (Fig. 6).

Pathway enrichment of static 1 Hz, 4 ms vs cells differentiated under ramped duration found significant (padj < 0.05) positive enrichment of metabolic processes, including fatty acid metabolism and lipid catalysis. Comparing 1 Hz, 4 ms to cells differentiated under ramped frequency stimulation, we found no significant pathway enrichment (supplementary Data S6–S7).

ClusterMap grouped specific GOBP Cardiac Conduction pathway genes irrespective of static/dynamic regimen. Compared to no stimulation controls and ramped frequency, cells differentiated under 1 Hz, 4 ms, 1 Hz, 8 ms, and ramped duration had upregulated the expression of calcium channel subunit *CACNB2* and transcription factor *NKX2-5*.^41,42^ Calcium channel subunit *CACNA1G* is upregulated in cells subjected to static 1 Hz, 4 ms and 2 Hz, 4 ms compared to cells differentiated under ramped frequency (padj = 0.09, 0.179).^43^ While not significant (padj = 0.135), potassium channel *KCNE1* was upregulated in cells differentiated under 1 Hz, 4 ms compared to ramped duration. Similarly, *KCNA5* and *KCNJ3* had adjusted p-values of 0.32 and 0.25 and log-fold changes of −1.41 and −1.00, respectively, between cells differentiated under 2 Hz, 4 ms and ramped duration regimens (supplementary Data S5).^44,45^

## DISCUSSION

Herein, we manufacture and validate an electrical stimulation device capable of generating complex electrical stimulation regimens. Composed of off-the-shelf electronics and costing a total of less than $100, this device can generate monophasic, alternating monophasic and biphasic stimulation regimens that vary in frequency from 0 to 50 Hz and pulse duration from 2 ms to 1 s within a 10% error. We use this to investigate cellular responses to static vs dynamic stimulation during hiPSC-CM differentiation and identify the more impactful electric stimulation parameter between pulse duration and frequency. We show that dynamic electrical stimulation improves cardiac differentiation efficiency, synchronicity, and intracellular calcium handling and flow but impedes contraction compared to static electrical stimulation and no stimulation controls. We also demonstrate that pulse duration is equally, if not more, critical as a stimulation parameter for optimizing hiPSC-CM differentiation and functionality. Across nearly all measured metrics, hiPSC-CMs subjected to dynamic pulse duration stimulation during differentiation outperformed those generated under dynamic frequency stimulation. Prior work independently examines the effect of constant electrical stimulation during differentiation^23^ or ramped frequency stimulation post differentiation^12^ but fails to recapitulate dynamic electrical current changes measured during embryogenesis. Our approach, including developing an open-source, low-cost electrical stimulation system that allows for dynamic frequency and/or pulse duration stimulation, allows cells to be subjected to electrical stimulation during differentiation. Our open-source instrumentation allows for the input of custom stimulation regimens varying both stimulation frequency and pulse duration over time and promotes future innovation and augmentation of electrical stimulation systems.

Our results on promoting cardiac differentiation via electric stimulation are well supported. Numerous stimulation regimens performed on differentiating PSCs result in increased CM purity and spontaneously beating clusters.^20,21,23,46^ Notably, we see non-significant increases in CM purity when cells are differentiated under dynamic regimens vs static conditions. We note that other studies stimulate cells only for specific hours of each day during hiPSC-CM differentiation,^23,46–48^ whereas we stimulate continuously, 24 h a day, throughout cardiac differentiation. Despite these differences in the study design, electrical stimulation, independent of regimen, is seen to enrich hiPSCs-CM difference efficiency.

In our optical assessment of hiPSC-CM calcium handling, we see the strongest impact of regimen type (dynamic vs static). As calcium flow in cardiomyocytes is intricately tied to their voltage membrane potential, we hypothesized that dynamic electrical stimulation would further mature hiPSC-CM calcium handling over static stimulation. We assessed calcium handling at various length scales, looking at intracellular flow, reuptake, and synchronicity. We assessed hiPSCs-CM calcium cycle FWHM (to measure the duration of elevated calcium levels and estimate the function of SERCA pump proteins) and tau (the decay constant of the calcium cycle to estimate the reuptake rate of sarcoplasmic reticulum). Furthermore, we quantified beating synchronicity and calcium flow velocity and directionality to assess intercellular hiPSC-CM connection and the formation of the cardiac syncytium. hiPSC-CM maturity is generally associated with longer FWHM, smaller decay constant, higher levels of synchronicity, faster calcium flow velocities, and more unidirectional calcium flow.^9,49^

Our data show that electrical stimulation during differentiation significantly improves beating synchronicity, as indicated by the lower TPA-MADs (Time of Peak Arrival Median Absolute Deviation) across all EStim groups compared to controls [Fig. 3(b)]. However, the effects on calcium handling parameters such as calcium cycle duration (FWHM) and decay constant (Tau) were variable and did not display a consistent trend across different stimulation regimens, except for the ramped duration group, which exhibited more consistent improvements [Figs. 3(c) and 3(d)].

Furthermore, we observed a depletive effect of EStim on hiPSC-CM contraction and relaxation velocities. Cells differentiated under electrical stimulation, across all regimens, showed significantly decreased contraction and relaxation velocities compared to no stimulation controls (Fig. 5). This effect was more pronounced in dynamic stimulation regimens than in static ones. This paradox suggests that while electrical stimulation enhances certain electrophysiological properties, such as synchronicity and, in some cases, calcium handling, it may concurrently impair mechanical properties like contraction velocity.

Two possible explanations may exist for this phenomenon. One pertains to the connection between non-CMs and hiPSC-CM electromechanics. Prior work demonstrates that high-purity hiPSC-CM cultures exhibit faster calcium flow but decreased contraction and relaxation velocity, demonstrating the impact that non-CMs have on hiPSC-CM function.^50^ As our study shows, electrical stimulation improves hiPSC-CM differentiation efficiency; the diverging functionality in calcium handling and contraction may be a result of lower amounts of non-CMs in the heterogeneous cell population. Furthermore, the composition of the remaining non-CMs in the heterogeneous cell culture may also be involved as cardiac fibroblasts^51^ and endothelial cells^52^ are shown to heavily influence electrochemical coupling and contraction. Our analysis here focuses on only hiPSC-CMs. Thus, further investigation of the resulting cell population after differentiation will be needed to rule out the role of the non-CMs in this diverging phenomenon. Another explanation may pertain to the specific cardiac subtypes generated under electrical stimulation. The contractile and calcium handling capabilities of cardiomyocytes differ based on location^53,54^ (atrial vs ventricular) and role^55^ (contractile vs conductive). In-depth characterization demonstrates significant differences in the conduction velocity and contraction velocity of ventricular cardiomyocytes and Purkinje fibers.^55,56^ Therefore, the diverging calcium handling and contraction detected in this study may be due to hiPSC-CM subtype specializations as has been recently shown.^24^ hiPSC-CM subtype analysis may be conducted to explore this further.

Finally, in our RNA sequencing analyses, we observed EStim to have a significant impact on the expression of genes that play key roles in cardiac development and function. Examining these analyses on the gene level indicates that electrical stimulation during cardiac differentiation may have a positive impact on differentiation outcomes. Higher *HCN4* expression was observed in the ramped frequency conditions, suggesting improved functionality of hiPSC-CMs in these groups, as *HCN4* has been found to play a critical role in maintaining normal cardiac rhythm in hiPSC-CM models and in establishing pacemaker action potentials in the murine embryonic heart.^37,57^ hiPSC-CMs differentiated under ramped conditions also developed higher expression of *BMP10*, with BMP10 growth factor generated by the *BMP10* gene playing a pivotal role in heart development as well as demonstrating cardioprotective properties.^38,58^ Ramped duration stimulation was observed to also increase the expression of *FTH1* and *FTL*, genes responsible for producing the heavy and light chains, respectively, of the iron storage and release protein ferritin. Ferritin is responsible for maintaining homeostatic iron levels, with low ferritin associated with elevated chance of cardiomyopathies.^59,60^ Although excessive ferritin resulting in iron accumulation can be detrimental to cells,^61^ the hiPSC-CMs differentiated under ramped duration stimulation did not display greater cell death or loss in functionality. As such, the increased ferritin production in conjunction with the improved calcium handling metrics of ramped duration hiPSC-CMs and the absence of known means for electrical stimulation in and of itself to stimulate ferritin production suggest that the greater *FTH1* and *FTL* expression is indicative of hiPSC-CMs differentiated under ramped duration being more functionally competent.

Greater *CORIN* expression relative to the control condition was also observed in the ramped duration, 1 Hz, 4 ms, and 1 Hz, 8 ms conditions. *CORIN* is highly expressed in the heart, is vital for maintaining normal blood pressure,^62^ and has even demonstrated cardioprotective effects,^63^ with *CORIN* knockout and low corin protein levels increasing the likelihood of hypertension and heart failure.^40,64–66^ Ramped duration, 1 Hz, 4 ms, and 1 Hz, 8 ms conditions were also observed to display a shared response to EStim in their expression of *CACNB2* and *NKX2-5*, which was upregulated for all three regimens. *CACNB2* encodes a subunit of calcium channels essential for signal transduction,^67^ and *NKX2-5* is a critical regulator of cardiac development and hallmark indicator of cardiomyocyte fate.^68,69^ As such, the ramped duration, 1 Hz, 4 ms, and 1 Hz, 8 ms regimens were observed to result in genetic changes indicative of more physiologic hiPSC-CMs. Conversely, the ramped frequency and 2 Hz, 4 ms stimulation conditions induced downregulation of *CORIN*, suggesting higher frequency stimulation had some detrimental impact on hiPSC-CM differentiation.

Most notably, the ramped duration condition was observed to induce the most significant differential gene expression indicative of more functional and physiologic hiPSC-CMs, which was in agreement with the observation that only the ramped duration stimulation condition yielded more consistent improvements in analyzed metrics.

Some limitations of the study exist, which warrant acknowledgment. Monolayer hiPSC-CM was assessed here rather than 3D cultures, which may limit the translation of the findings to the clinic, as 3D assembly of hiPSC-CMs is seen to increase functionality and maturation,^12,70^ and 3D constructs may modulate electric charge propagation.^71^ Due to the complexity of 3D differentiation schemes, coupled with lower efficiency and reproducibility issues seen with these schemes,^72–76^ monolayer differentiation was selected for this early study. Importantly, we wanted to ensure all cells are exposed to the same electrical stimulation condition, which may not be the case for 3D cultures. The open-source nature of the device encourages future applications with 3D systems. Additionally, the initiation of electrical stimulation and differentiation was timed to commence and terminate simultaneously. Recent work has revealed the importance of parameters like frequency and onset during the differentiation scheme on the subtype of the hiPSC-CMs produced.^24,25^ Future work may investigate the covariance of both onset and pulse frequency over differentiation easily with this experimental setup.

In summary, this work produces and validates a practical and inexpensive electrical stimulation simulation device. We show that our device can reproducibly generate electrical stimulation regimens capable of influencing cellular behavior, as demonstrated by our deployment of the device during hiPSC-CM differentiation changing differentiation yields, enhancing beating synchronization, and changing calcium handling and beating velocity. We highlight the significant impact of our device's control over stimulation pulse duration, particularly in dynamic regimens, on hiPSC-CM differentiation and functionality, identifying it as a potential area for future optimization. Finally, we release to the community an open-source, low-cost electrical stimulation system to increase access to our methodology and to encourage further research into electrical stimulation's role in hiPSC-CM function and maturation.

## METHODS

### Device synthesis and validation

**FIG. 7.**
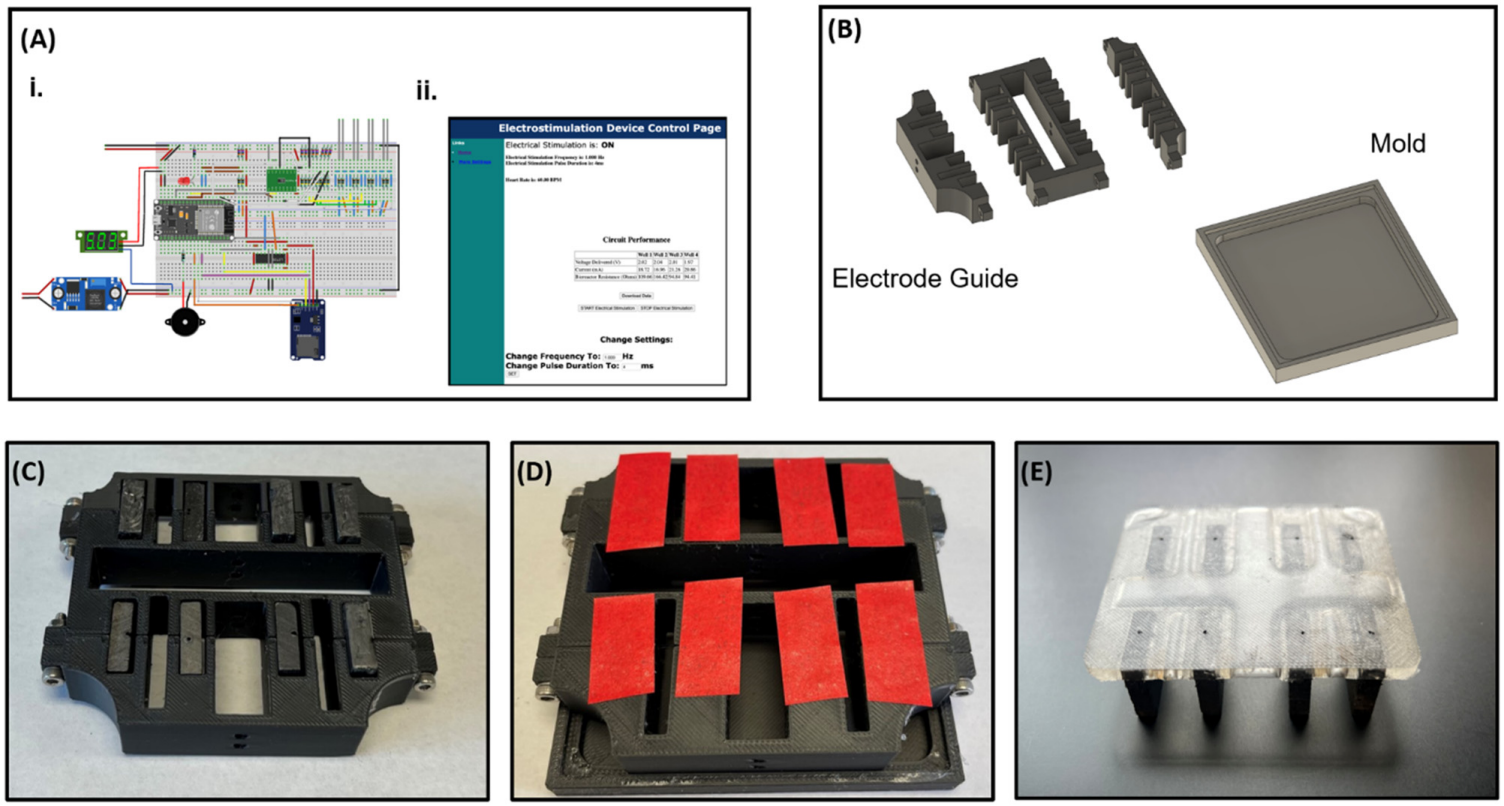
Electrical stimulation device design and manufacturing (a): (i) Electrical stimulation voltage-sensing circuit. (ii) Web server that manages stimulation parameters and enables downloads of stimulation data. (b) Two-part electrode building mold, with electrode guide and PDMS mold. (c) Electrodes loaded into electrode guide. (d) Mold with PDMS poured and electrodes secured into guide with tape. (e) Final electrodes with wire holes drilled.

The low-cost electrical stimulation device is comprised of two parts: (1) a signal generator and (2) in-well electrodes. The signal generator is assembled from an Arduino ESP32 microcontroller, L293D motor h-bridge, and LM2596 high voltage buck converter [Fig. 7(a)]. The in-well electrodes comprise graphite carbon blocks cast in polydimethylsiloxane (PDMS). Complete fabrication instructions and program code can be found in the supplementary material. Custom molds are 3D printed with temperature-resistant filament [Fig. 7(b)]. Carbon-graphite blocks are then cut and secured in molds [Fig. 7(c)]. PDMS (1:10 elastomer/crosslinker ratio) is poured into a mold to secure carbon blocks at specific spacing for 6-well plates [Fig. 7(d)]. After PDMS curing, holes are drilled through the PDMS and into the carbon blocks for electrode pin insertion [Fig. 7(e)]. Electrode pins are connected to the pulse generator. The signal generator is programmed via custom Arduino code to generate biphasic electrical signals, measure/record voltage, current, and resistance, and connect to a wireless graphic user interface (GUI). Signal generation was validated across a range of voltages and pulse durations via oscilloscope recording (Tektronix). Percent errors from expected values were calculated for each measured setting. Pulse sensing module, which leverages the built-in ESP32 analog-digital converter, was assessed and calibrated using an oscilloscope and a test circuit with known voltage and resistance. Biocompatibility of electrical stimulation was confirmed via LIVE/DEAD staining (Proteintech, PF00007) and staining for pluripotency markers after 24 and 72 h of stimulation at tested settings (4 V/4 ms/1 Hz, 4 V/4 ms/2 Hz, and 4 V/8 ms/1 Hz). The percentage of live cells was calculated by dividing the number of cells positively stained for Calcein AM (live) by the total cell number, calculated as the sum of positively stained for Calcein AM (live) and ethidium homodimer (dead).

### Cardiac differentiation and electrical stimulation

WTC-11 hiPSCs, transfected to express CMV-GCaMP2, a gift from Dr. Bruce Conklin, were seeded on Matrigel-coated plates (0.1 mg/ml) at 28 000 hiPSCs per cm^2^. Cardiac differentiation followed a modified protocol of Wnt pathway modulation.^77^ Cells were cultured in Essential 8 (E8) media for 72 h, with media changes every 24 h. On day 0 of cardiac differentiation, E8 media was replaced with RPMI 1640 media with B27 no insulin (RB-) and 12 *μ*M CHIR99021. After 24 h, cells were transitioned to RB- media for 48 h. On day 3 of differentiation, Wnt signaling was inhibited by incubating cells in RB- media with 5 mM IWP2 (inhibitor of WNT production). 48 h after inhibition with IWP2, cells were allowed to recover in RB- for another 48 h and in CDM3 media [RPMI 1640 with h-albumin (0.5 mg/mL) and L-ascorbic acid 2-phosphate (0.213 mg/mL)] thereafter. Electrical stimulation was also initiated on day 0 of cardiac differentiation. Cells were stimulated continuously from day 0 to day 10 of cardiac differentiation along 5 conditions (Voltage/Pulse Duration/Frequency). Three conditions were static over the 10 days, 1 Hz, 4 ms (4 V/4 ms/1 Hz), 2 Hz,4 ms (4 V/4 ms/2 Hz), and 1 Hz, 8 ms (4 V/8 ms/1 Hz), while 2 were dynamic over the 10 days of stimulation. Dynamic frequency stimulation, termed ramped frequency stimulation, followed a linear increase in frequency from 1 to 2 Hz at 6.94 ×10^−5^ Hz/min (equivalent to 0.1 Hz per day). Dynamic pulse duration stimulation, termed ramped duration stimulation, followed a similar linear pulse duration from 4 to 8 ms at 2.77 ×10^−5^ ms/min (equivalent to 0.4 ms per day). Starting and ending parameters of dynamic regimens were set according to consultation with prior work. Prior work has demonstrated support for the positive benefits of stimulation at both 1 Hz^23,47,48,78–81^ and 2 Hz.^82–84^ In dynamically ramping between these two set points over 10 days, the length of the differentiation protocol used here, we achieve 0.1 Hz/day. Similarly, prior work supports stimulation with a pulse duration of 4 ms,^11,23,85^ the shortest pulse duration we may achieve with minimal error. Notably, cell apoptosis is directly correlated with longer pulse duration.^86^ Therefore, 8 ms was found to be the longest pulse duration that did not result in significant cell apoptosis after 72 h of continuous stimulation, the longest time between cell media changes in the cardiac differentiation protocol employed. Ramping pulse duration from 4 to 8 ms over 10 days yields a rate of 0.4 ms/day.

### Immunostaining and image processing

Cells differentiated under EStim and no stimulation controls were fixed and stained on day 10 to assess functionality and cardiac purity. Cells were fixed with 4% paraformaldehyde (Polysciences) for 10 min at room temperature (R/T), rinsed with 300 mM glycine (Sigma-Aldrich), and permeabilized with 0.2% Triton X-100 (Amresco). Cells were then incubated in blocking buffer, containing 1% bovine serum albumin (BSA) and 0.1% Tween-20 (Fisher Scientific) for 30 min and incubated overnight at 4 °C with primary antibody in blocking buffer. Tested primary antibodies included cardiac troponin T (cTnT; 1:500, Proteintech, 15513-1-AP) and Octamer-binding transcription factor 4 (OCT-4; 1:40, Invitrogen, MA5-14845). Cells were then washed with blocking buffer and incubated with anti-rabbit Alexa-488 secondary antibody in blocking buffer (1:400, Sigma-Aldrich, SAB4600044). Cells were co-stained with nuclear Draq5 (Cell Signal, 4048L) or 4′, 6-Diamidine-2′-phenylindole dihydrochloride (DAPI; Thermo Fisher, D1306) stain. Stained samples were imaged using an Olympus FV3000 Confocal Laser Scanning Microscope. Cardiomyocyte differentiation efficiency was estimated via the ratio of cTnT-positive area to Draq5-stained area, measured with Fiji software, as described previously.^87^ Nuclei aspect ratio was assessed by using cTnT-positive areas as a mask on Draq5 positive areas and calculating, via Fiji, the aspect ratio of CM-specific nuclei. cTnT fiber orientation was assessed via ImageJ plugin OrientiationJ, as previously described.^88^

### Intracellular calcium transients imaging

The flow of intracellular calcium was facilitated via high-speed imaging of CMs differentiated from CMV-GCaMP2 transfected hiPSCs. On day 10 of cardiac differentiation, GCaMP cells were imaged on a Leica DMI6000 B microscope and a 10X Leica objective at 20 frames per second. With the imaging setup utilized, 20 frames per second provided the best balance of fluorescent signal-to-noise ratio and temporal resolution to capture all contractions of our samples, as indicated by our ability to generate fluorescent signal traces from the shortest CM contractions, and similar image capture rates have been utilized and validated in our previous work.^89^ The cyclic intensity of green fluorescent protein (GFP), representing the intracellular concentration of calcium, was captured in image sequences of 120 images. Images were processed using a custom MATLAB code to assess the degree of synchronicity, calcium cycle duration, and decay constant, as previously discussed.^89^ Images were also processed with the Optical Flow Analysis Toolbox in MATLAB for Mesoscale brain activity (OFAMM) to derive calcium flow directionality and velocity.^90^ Briefly, image sequences were preprocessed using median spatial filters (3 × 3 pixels) and low pass (<1 Hz) temporal filters as recommended. Processed image sequences were then passed to the toolbox, wherein the Horn-Schunck (HS) optical flow method was employed to calculate flow vectors on a pixel-by-pixel basis. Vector directionality was assessed by calculating the circular variance of the vector phase, whereas velocity was calculated as vector magnitude.^91^

### Contractility assessment

Phase contrast images of hiPSC-CMs were taken on a Leica DMI6000 B microscope using a 10X Leica objective at 20 frames per second. Images were processed using BeatProfiler, an open-source optical flow program that can quantify contractile function and force generation.^92^ Derived contraction velocity metrics were averaged across all peaks and normalized to image length scales.

### RNA sequencing and analysis

Total RNA was collected from separate differentiations, purified with RNeasy Mini Kit (Qiagen) according to the manufacturer's protocols, and submitted to the University of Texas Genomic Sequencing and Analysis Facility (Center for Biomedical Research Support, RRID: SCR 021713). Tagseq libraries were sequenced using the NovaSeq 6000 SR100 and preprocessed by the Bioinformatics Consulting Group at the University of Texas at Austin. Reads were then processed using the nf-core RNAseq pipeline.^93,94^ Briefly, reads were aligned to human reference genome GRCh38 using “STAR,” before quantifying gene counts using “Salmon.”^95,96^ Differentially expressed genes were determined using the edgeR's quasi-likelihood method.^97^ Differentially expressed genes were defined to have an adjusted p-value <0.05 (Benjamini-Hotchberg) and log-fold-change of ±1. Genes, ranked by log-fold-change, were used for gene-set enrichment analysis with fgsea package^98^ in R and the Gene Ontology Biological Processes (GOBP) set.^99,100^ Significantly enriched pathways were defined as having adjusted p-values <0.05. Gene counts and associated code can be found at https://github.com/ZoldanLab/Estim.

### Statistical analysis

Statistical analysis was conducted using a one-way analysis of variance test and post hoc Tukey's multiple comparison tests using Prism 9 software (GraphPad). Relationships were deemed significant at a threshold of p < 0.05. The data are presented as mean values ± standard error. Statistical tests were performed after Robust regression and Outlier removal (ROUT) at Q = 1% (GraphPad).

## SUPPLEMENTARY MATERIAL

See the supplementary material for additional details on the validation of the signal generator (Fig. S1), pulse sensing module (Fig. S2), dynamic pulse parameter feature (Fig. S3), cTnt Orientation Data (Fig. S4), differential expression data (Fig. S5), pathway enrichment data (Figs. S6 and S7), and device manufacturing instructions (Fig. S8). Representative videos of hiSPC-CM GCaMP signal, used in the assessment synchronicity and calcium flow, are also attached as Video 1 (No Stim.), Video 2 (1 Hz, 4 ms), Video 3 (2 Hz, 4 ms), Video 4 (1 Hz, 8 ms), Video 5 (Ramped Frequency), and Video 6 (Ramped Duration).

## Data Availability

The data that support the findings of this study are available from the corresponding author upon reasonable request.
